# Spatiotemporal Observation of Monosodium Urate Crystals Deposition in Synovial Organoids Using Label-Free Stimulated Raman Scattering

**DOI:** 10.34133/research.0373

**Published:** 2024-05-27

**Authors:** Yaxin Chen, Ziyi Chen, Wenjuan Wang, Yinghui Hua, Minbiao Ji

**Affiliations:** ^1^ State Key Laboratory of Surface Physics and Department of Physics, Human Phenome Institute, Academy for Engineering and Technology, Key Laboratory of Micro and Nano Photonic Structures (Ministry of Education), Shanghai Key Laboratory of Metasurfaces for Light Manipulation, Fudan University, Shanghai 200433, China.; ^2^ Department of Sports Medicine, Huashan Hospital, Fudan University, Shanghai, China.

## Abstract

Gout, a common form of arthritis, is characterized by the deposition of monosodium urate (MSU) crystals in joints. MSU deposition in synovial tissues would initiate arthritis flares and recurrence, causing irreversible joint damage. However, the dynamic deposition of MSU crystals in tissues lacks experimental observation. In this study, we used chemical-specific, label-free stimulated Raman scattering (SRS) microscopy to investigate the spatiotemporal deposition and morphological characteristics of MSU crystals in human synovial organoids. Our findings revealed a critical 12-h window for MSU deposition in the lining layer of gouty synovium. Moreover, distinctive inflammatory reactions of the lining and sublining synovial layers in gout using SRS microscopy were further verified by immunofluorescence. Importantly, we identified a crucial proinflammatory role of sublining fibroblast-like synoviocytes, indicating a need for targeted medication treatment on these cells. Our work contributes to the fundamental understanding of MSU-based diseases and offers valuable insights for the future development of targeted gout therapies.

## Introduction

Gout, a prevalent form of inflammatory arthritis, is triggered by the deposition of monosodium urate (MSU) crystals in joints [1]. The global incidence of gout has been on the rise due to changes in dietary habits, increased life expectancy, and the obesity epidemic [2]. The initial stage of gout involves the deposition of MSU crystals in the synovium, which subsequently activates resident macrophage-like synoviocytes or fibroblast-like synoviocytes (FLS) [3–5]. This activation then leads to the recruitment of immune cells [6]. FLS also mediates inflammatory tissue priming promoting site-specific recurrence of gout [7]. Despite these findings, there is a dearth of in-depth studies on the deposition of MSU crystals in gouty synovial tissues and their interaction with FLS.

Nonlinear optical microscopy leverages the optical properties of endogenous compounds to offer chemical contrasts, enabling intrinsic 3-dimensional (3D) visualization of structures without the need for exogenous labeling. Specifically, coherent Raman scattering techniques, such as coherent anti-stokes Raman scattering and stimulated Raman scattering (SRS), provide label-free chemical detection of compounds based on their intrinsic vibrational signatures [8–10]. Over the past decades, SRS-based methods have been employed for refined imaging in various biomedical applications [11–13]. Our team has previously demonstrated the highly specific and label-free imaging capability of MSU crystals in sections of gouty tissues using SRS [11–14]. However, we were unable to dynamically track the in situ deposition of MSU crystals. By exploiting the translucent nature of synovial organoids and the 3D chemical imaging capability of SRS microscopy, we are able to observe the spatiotemporal deposition process of MSU crystals in unlabeled tissues. These approaches also ensure the homogeneity of the experiments.

In this study, we used SRS microscopy to initially uncover the spatiotemporal deposition and morphological characteristics of MSU crystals in synovial organoids. We found that a 12-h period may be the critical window for substantial deposition of MSU crystals in the lining layer of gouty synovium. Further investigations into the responses of lining and sublining FLS using SRS microscopy and immunofluorescence revealed a substantial proinflammatory role of sublining FLS in gout.

## Results

**Fig. 1.**
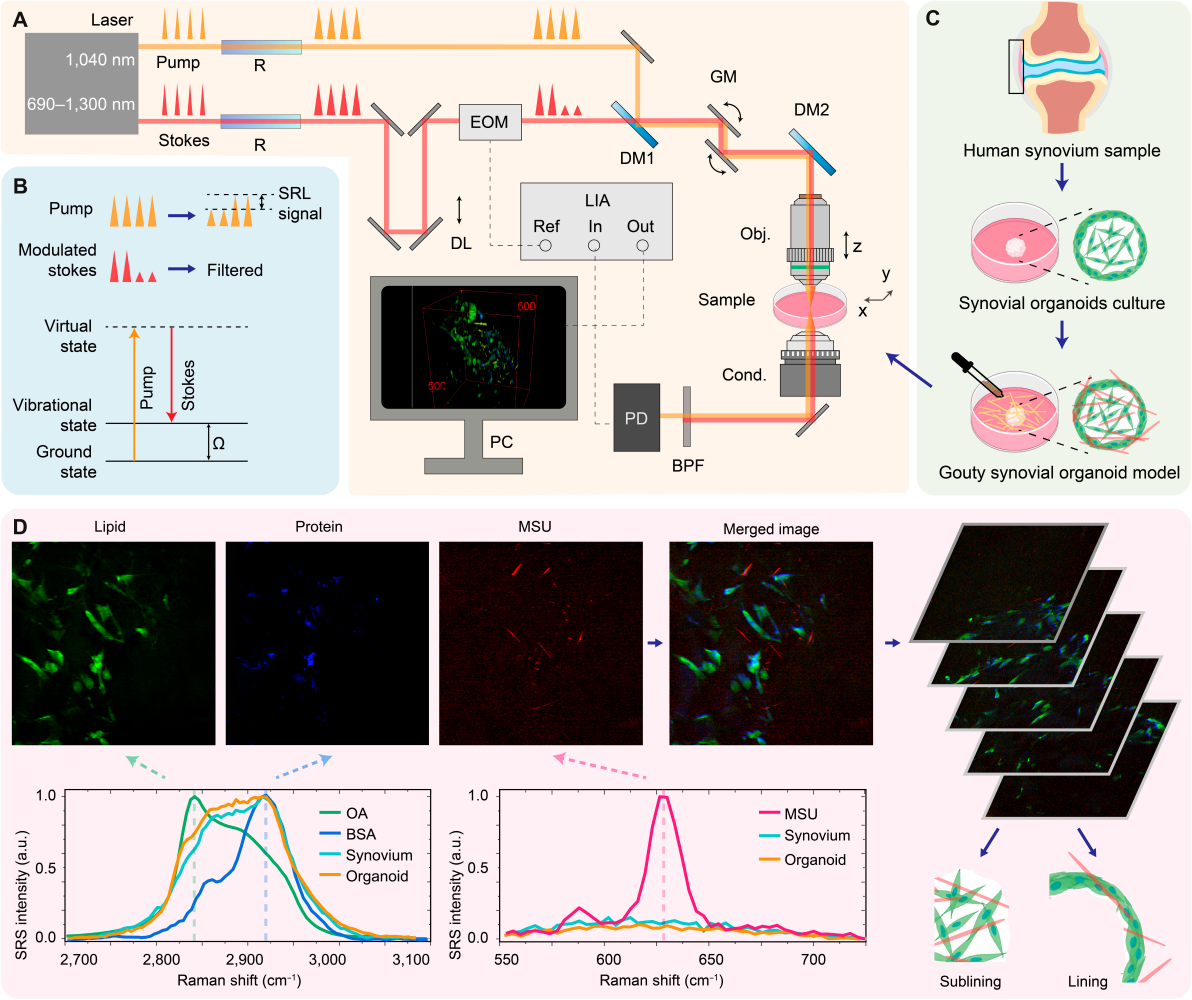
Overview of the experimental procedure. (A) Experimental setup: Illustration of SRS signal detection. The femtosecond pump and stokes beams were elongated by 2 SF57 glass rods to achieve spectral resolution. The coherent pump and stokes beams were introduced to the sample, inducing stimulated emission. The modulation of the stokes beam was transferred to the pump beam and demodulated by a lock-in amplifier. The abbreviations used are as follows: R, SF57 glass rod; DL, delay line; EOM, electro-optical modulator; DM, dichroic mirror; GM, galvo mirror; BPF, band-pass filter; PD, photodiode; PMT, photomultiplier tube; LIA, lock-in amplifier. (B) Energy diagram and detected process: In the SRS process, the pump and stokes beams interact with the organoid sample simultaneously. When the beating frequency matched the Raman vibrational mode Ω, a coherently amplified energy transfer process occurred, resulting in the annihilation of the pump photon (SRL) and the generation of the stokes photon (stimulated Raman gain). In our experiment, the stimulated Raman gain signal was filtered out, and the SRL signal was detected. (C) Organoid cultivation: Human synovial tissue was harvested from patients with meniscus injuries through arthroscopic surgery. Synovial organoids were established after a 21-d culture period. Subsequently, MSU crystals were added to the organoids to create an acute-gout synovial organoid model. (D) Data collection and processing: The appropriate pump and stokes wavelength combination was selected for imaging lipids, proteins, and MSU crystals in the synovium organoid. In situ images of lipids and proteins were decomposed according to the SRS spectra displayed in the lower panel. By moving the objective along the z-axis, 3D information about the organoid was acquired, and lining and sublining layers could be differentiated based on cell density. ImageJ and MATLAB were then used for data processing. The abbreviations used are as follows: OA, oleic acid; BSA, bovine serum albumin.

The experimental design is depicted in Fig. 1. Figure 1A illustrates our home-built SRS microscopy setup. Figure 1B illustrates the energy diagram of the SRS process, where the pump and stokes beams simultaneously interact with the sample at the matched Raman frequency. Human acute-gout synovial organoids were cultured for 21 d and then stimulated with MSU crystals (Fig. 1C). We then obtained SRS images at 630 cm^−1^ to specifically identify MSU crystals, while those at 2,845 and 2,930 cm^−1^ represented cellular lipid and protein distribution (Fig. 1D). Figures S1 to S3 present the spectral and spatial resolutions as well as the spectral width.

### Synovial organoid establishment and 3D reconstruction using SRS microscopy

**Fig. 2.**
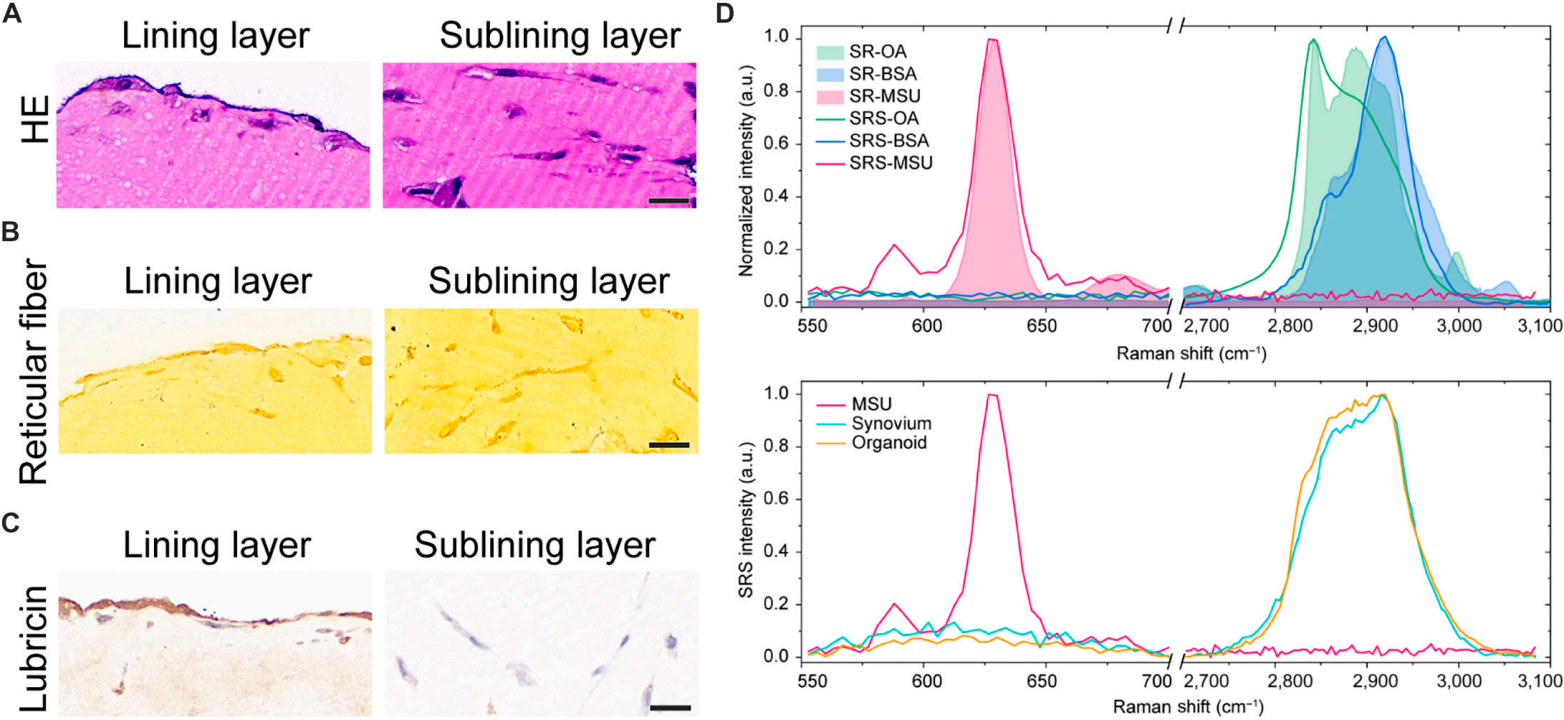
Establishment of synovial organoids. (A to C) HE, reticular fiber, and lubricin IHC staining of human 3D synovial organoids. The left panel shows the lining layer, and the right panel shows the sublining layer. Scale bar: 20 μm. (D) Spontaneous and SRS analysis of standard OA, BSA, and MSU (upper panel) and lower panel presents the SRS spectra of MSU, synovium tissue, and synovium organoid. The SRS spectra were consistent with the spontaneous Raman (SR) spectra and had characteristic features that distinguished MSU crystals in the synovium organoid. The synovium organoid exhibited similar SRS spectra to fresh synovium tissue.

To evaluate the synovial organoids, we initially performed standard histological staining methods, including hematoxylin–eosin (HE) staining, reticular fiber staining, and immunohistochemistry (IHC). The histological examination revealed characteristic lining and sublining layers in the synovial organoids (Fig. 2A and B). Immunoreactive lubricin was predominantly detected in the lining layer of the organoids, with a sparse presence in cells beneath the lining layer (Fig. 2C). Subsequently, SRS spectral imaging was employed to characterize the gout synovial organoids. Two specific spectral regions were selected to characterize the vibrational signatures of synovial tissue, organoids, and MSU crystals. The lower-frequency region of 550 to 700 cm^−1^ exhibited the Raman peak of the purine ring breathing vibrational mode of MSU molecules, with lipid and protein (represented by standard oleic acid and bovine serum albumin) exhibiting clean Raman backgrounds (Fig. 2D). The higher-frequency region of 2,700 to 3,100 cm^−1^ predominantly revealed lipid and protein contents, with protein showing high-intensity CH_3_ (2,930 cm^−1^) and low-intensity CH_2_ (2,845 cm^−1^) vibrations and lipid displaying high intensities at both Raman frequencies, as depicted in the upper panel of Fig. 2D. SRS spectra of cultivated organoids and fresh synovium tissue exhibited similar spectral features, indicating a mixture of lipid and protein (the lower panel of Fig. 2D). These combined spectral and histological characterization findings demonstrated that the structure and function of synovial organoids closely resembled those of synovial tissue, signifying the successful establishment of synovial organoids.

**Fig. 3.**
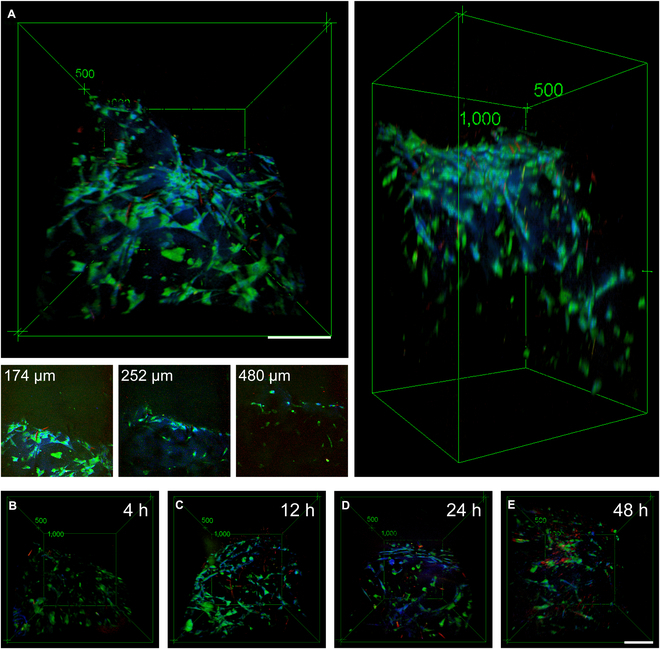
3D reconstruction using SRS microscopy. (A) Top, side, and sectional views at different depths of the organoid (left upper, right, and left lower panels, respectively) after MSU crystal supplementation for 6 h. Scale bar: 100 μm. (B to E) Top views of the organoid after MSU crystal addition for 0, 12, 24, and 48 h, respectively. MSU crystals (red, 630 cm^−1^), lipid (green, 2,930 cm^−1^), and protein (blue, 2,930 cm^−1^). Scale bar: 100 μm.

The 3D image stacks of acute-gout synovial organoids were captured via SRS imaging without the need for labeling. Figure 2E showcases the top view, side view, and sectional views at various depths of the synovial organoids following stimulation with MSU crystals for 6 h. Additionally, Fig. 3A to E presents the top views of the synovial organoids stimulated by MSU crystals for 0, 12, 24, and 48 h, respectively (Fig. S4).

### Temporal deposition of MSU crystals in synovial organoids

**Fig. 4.**
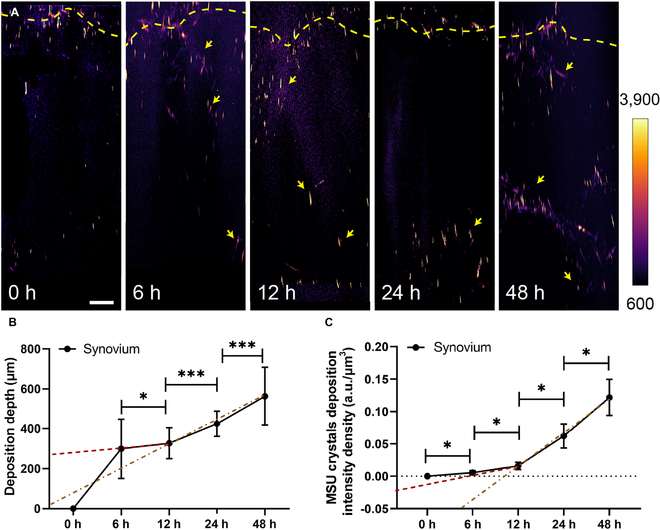
Temporal deposition of MSU crystals in synovial organoids. (A) Heatmap illustrating MSU crystal intensity in synovial organoids after 6, 12, 24, and 48 h of MSU crystal exposure. Yellow dashed lines denote the organoid surface, while yellow arrows indicate areas with MSU crystals. Scale bar: 100 μm. (B) Distribution of MSU crystals in synovial organoids at 0, 6, 12, 24, and 48 h. (C) Intensity density of MSU crystal deposition in synovial organoids at 0, 6, 12, 24, and 48 h. * represents *P* < 0.05, and *** indicates *P* < 0.001.

The deposition process of all MSU crystals in acute-gout synovial organoids could be visualized by the side views at varying time points (Fig. 4A). We observed that the overall density of the MSU crystals deposited in synovial organoids gradually increased over time, and the MSU deposition depth increased. To quantify our results, we extracted the MSU crystal deposition depth and intensity density inside the organoids (see Materials and Methods). We registered average MSU crystal sedimented depths of 299.8, 327.1, 425.0, and 563.3 μm at 6, 12, 24, and 48 h, respectively (Fig. 4B). Remarkably, the depth and intensity growth gradients were distinctly higher after 12 h, suggesting that it might represent the acceleration point for MSU crystal deposition in acute-gout synovial organoids (Fig. 4C).

### Spatial deposition of MSU crystals in synovial organoids

**Fig. 5.**
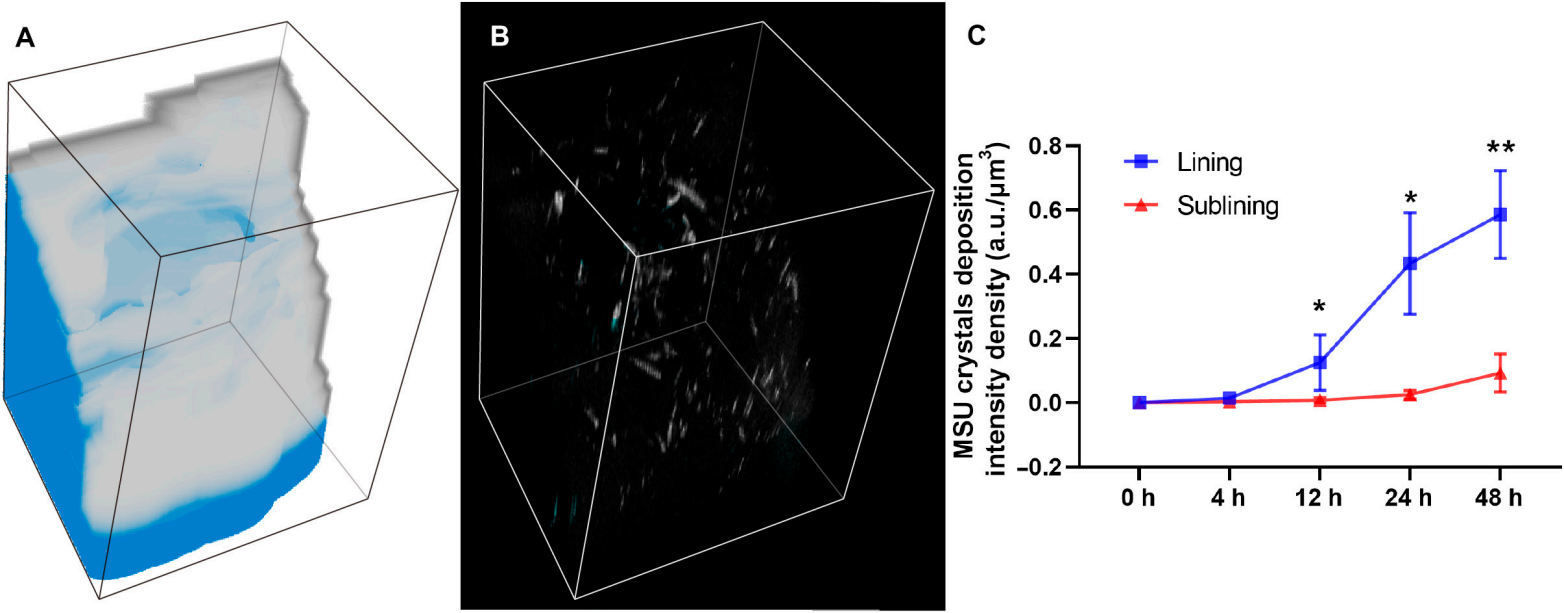
Spatial deposition of MSU crystals in lining and sublining layers. (A) Stereoscopic view showing the lining (white) and sublining (blue) layers of a typical field of view (FoV) measuring 508.93 × 508.93 × 915.68 μm^3^. (B) Stereoscopic view displaying MSU crystals in synovial organoids within the same FoV as (A), with MSU crystals marked in white and blue to represent deposition in the lining and sublining layers, respectively. (C) Intensity density of MSU crystal deposition in the lining and sublining layers of synovial organoids at 0, 6, 12, 24, and 48 h.

The synovial organoids, comprising lining and sublining layers, enabled separate visualization of MSU crystal deposition intensity density in these layers (Fig. 5A), as detailed in the Materials and Methods. A typical field of view (FoV) at 12 h post-MSU crystal addition is depicted in Fig. 5B, with crystals distinctly marked in the lining and sublining layers (white and blue, respectively). Notably, the growth kinetics of MSU deposition in both layers exhibited a similar acceleration point at 12 h, with a higher deposition rate observed in the lining layer compared to the sublining layer (Fig. 5C).

**Fig. 6.**
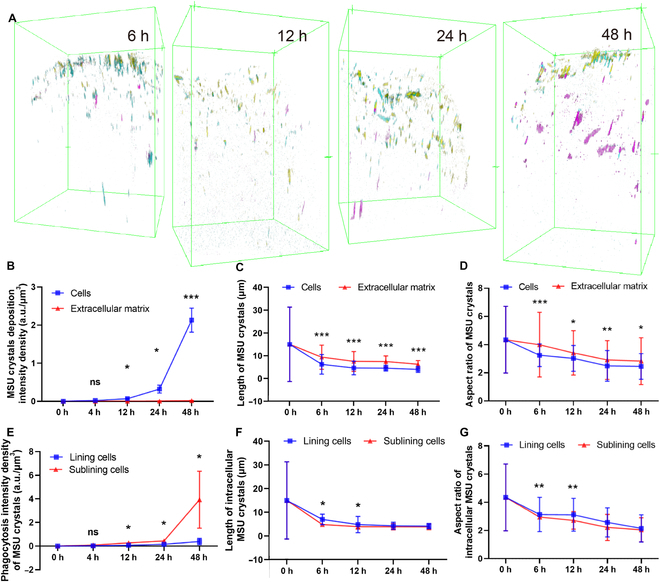
Spatial deposition of MSU crystals in cells and ECM. (A) Typical stereoscopic views of MSU crystals in synovial organoids at 6, 12, 24, and 48 h. MSU crystals in lining and sublining cells are marked in yellow and cyan, respectively, while those in the ECM are marked in magenta. (B to D) MSU deposition intensity density (B), average length (C), and aspect ratio (D) of MSU crystals in cells and ECM of synovial organoids at 0, 6, 12, 24, and 48 h. (E to G) MSU deposition intensity density (E), average length (F), and aspect ratio (G) of MSU crystals in lining and sublining cells of synovial organoids at 0, 6, 12, 24, and 48 h. * denotes *P* < 0.05, ** denotes *P* < 0.01, and *** denotes *P* < 0.001.

We further differentiated the intracellularly phagocytosed and the extracellularly deposited MSU crystals with the aid of 3D multichannel SRS imaging of MSU and lipid/protein in synovial organoids. Stereograms of MSU crystals in cells and extracellular matrix (ECM) are presented in Fig. 6A. Over time, both types of crystals exhibited increased aggregate deposition intensity densities, particularly after 12 h. However, the overall density and growth rate were notably higher for phagocytosed MSU crystals within cells compared to those in the ECM (Fig. 6B), underscoring the dominant role of phagocytosis in MSU uptake within synovial organoids.

To assess the crystal processing capability, we analyzed the spatiotemporal evolution of crystals in the cells and ECM. Results indicated a reduction in the length and aspect ratio of MSU crystals over time, with intracellular crystals being shorter in length and smaller in aspect ratio than those in the ECM (Fig. 6C and D), indicative of robust MSU degradation by organoid cells.

To compare the degradation abilities of MSU crystals between lining and sublining cells, we analyzed phagocytosed MSU crystals in both cell types. The intensity densities of MSU crystals phagocytized by sublining FLS were significantly larger than those by lining FLS after 12 h (Fig. 6E), indicating a more crucial role of sublining FLS in crystal degradation. Subsequently, we compared the morphological changes of intracellular MSU crystals in the lining and sublining layers at different time points. Similarly, the length and aspect ratio of MSU crystals in both cell types progressively decreased over time. MSU crystals in sublining cells were shorter in length and smaller in aspect ratio than those in lining cells from 6 to 12 h, suggesting a stronger ability of sublining cells to degrade crystals (Fig. 6F and G). These findings underscore the importance of interactions between sublining cells and MSU crystals, warranting further attention in gout studies.

### Responses of lining and sublining cells to MSU crystals

In our final set of experiments, we examined how synovial cells respond to MSU crystals, given the crucial role the synovium plays in gout. We employed SRS microscopy to monitor changes in 3D cell morphologies in real-time and used immunofluorescence to observe the cells’ inflammatory responses.

**Fig. 7.**
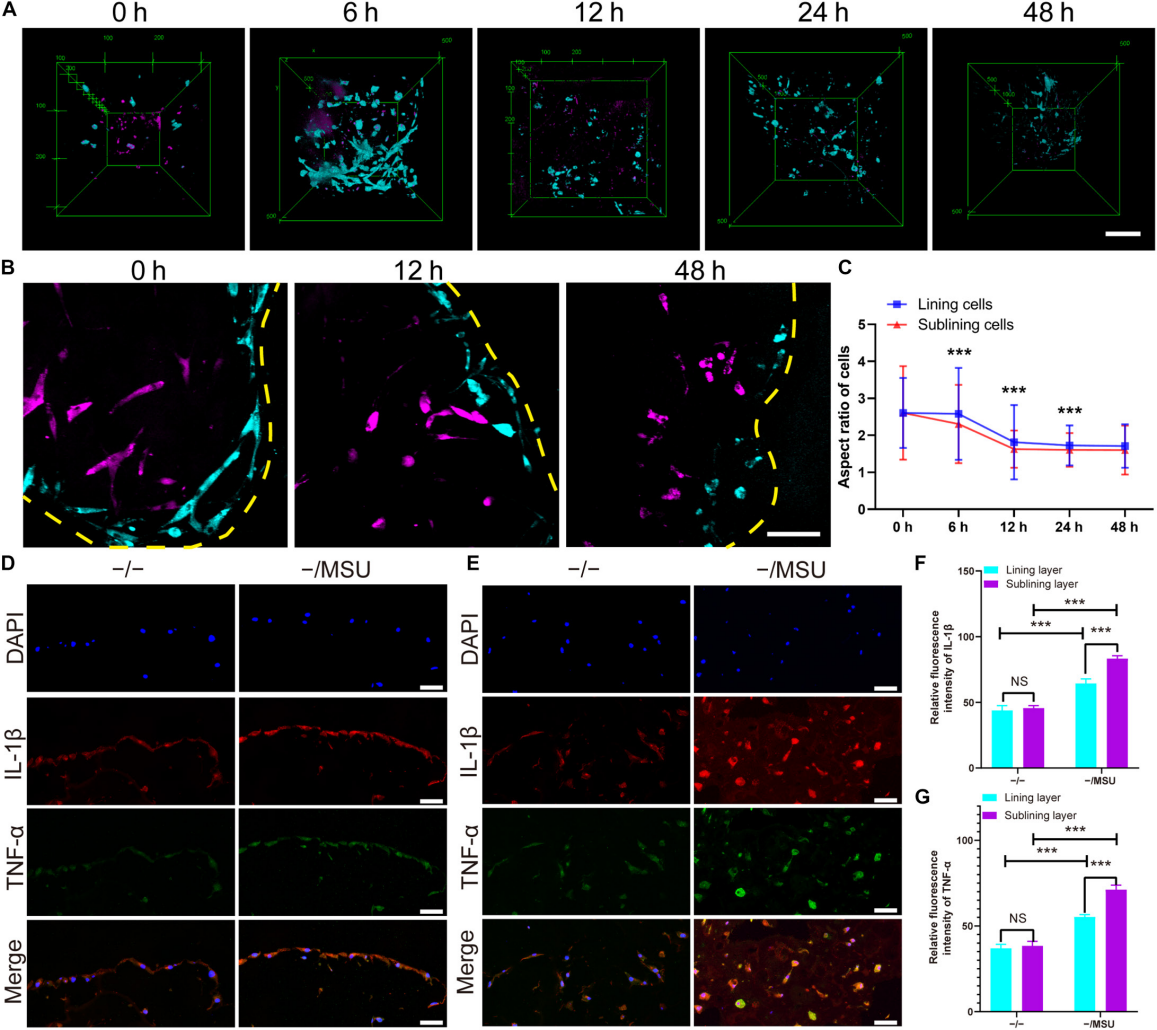
Response of lining and sublining cells to MSU crystals in synovial organoids. (A and B) Top views (A) and sectional views (B) of cells in acute-gout synovial organoids at 0, 6, 12, 24, and 48 h. Lining cells are marked in cyan, while sublining cells are marked in magenta. Yellow dashed lines indicate the organoid surface. Scale bar: 100 μm. (C) Aspect ratio of lining and sublining cells in acute-gout synovial organoids at 0, 6, 12, 24, and 48 h. (D and E) Representative images of double immunofluorescent staining of IL-1β and TNF-α in the lining (D) and sublining (E) layers of synovial organoids with or without MSU crystal addition. DAPI, 4′,6-Diamidino-2′-phenylindole. (F and G) Relative fluorescence intensity of IL-1β (F) and TNF-α (G) in the lining and sublining layers of synovial organoids with or without MSU crystal addition. Scale bar: 50 μm. ** denotes *P* < 0.01, and *** denotes *P* < 0.001.

We captured stereograms and typical sectional views of both lining and sublining cells at various time intervals following stimulation with MSU crystals (Fig. 7A and B). Over time, we observed that the deformation of both cell types became more pronounced, with an increase in roundness (Fig. 7C). Interestingly, the sublining cells appeared to be more rounded than the lining cells. Furthermore, immunofluorescence revealed the cellular inflammatory responses in both the lining and sublining layers, with and without MSU crystal stimulation (Fig. 7D and E). When compared to the group without MSU crystals, the synovial organoids stimulated by MSU crystals displayed stronger fluorescence intensities of inflammatory factors such as interleukin-1β (IL-1β) and tumor necrosis factor-α (TNF-α) (Fig. 7F and G). Notably, the relative fluorescence intensities in the sublining cells were higher than those in the lining cells. These findings suggest that sublining FLS may play a more critical role than lining FLS in mediating the inflammatory reactions during the progression of gout.

## Discussion

For years, gout patients have endured joint pain and loss of function. These issues have remained difficult to address due to the lack of effective treatment options. Previous researches have shown that synoviocytes, when stimulated by MSU crystals, can secrete proinflammatory mediators [15,16]. These mediators then activated chondrocytes and immune cells, intensifying synovial inflammation and oxidative and pain states [7,17]. The mechanisms by which MSU crystals interact with synovial tissues in gout have garnered increasing attention and are crucial for guiding future therapies. Our study has filled a gap in this area by investigating the dynamic deposition process of MSU crystals in synovial organoids using SRS imaging. We observed the spatiotemporal deposition and morphological characteristics of the crystals. Our research discovered that 12 h may be the window period for MSU crystals to largely deposit in the lining layer of gouty synovium. Additionally, we found a vital proinflammatory role for sublining FLS in gout.

Our results showed that the depth and density of crystal deposition in synovial organoids significantly increased after 12 h. The reason for the 12-h turning point might be the MSU crystal deposition from the synovial lining to the sublining. The high cell density and active cellular migration of the synovial lining layer result in higher resistance, slowing down the deposition. The low cell density and loose extracellular connective tissues of the synovial sublining layer lead to less resistance, thereby faster deposition. This result provided visual evidence that 12 h could represent the time window for MSU crystal deposition in the synovial lining layer, and that beyond 12 h the crystals deposited faster in the sublining layer.

To date, the key to gout treatment has been long-term urate-lowering therapy and anti-inflammatory agent use [18]. For gouty arthritis that occurred once with evidence of urate deposition in the joints, urate-lowering therapy should be started [19]. However, the ideal start for urate-lowering therapy is controversial. Our study simulated the first episode of acute gouty arthritis in the synovium and indicated that urate-lowering therapy could be started within 12 h of the onset to prevent massive crystals deposition in the tissues. Interestingly, the latest “Guideline for the Diagnosis and Treatment of Gout (2023)” formulated by the Chinese Rheumatology Association also recommended to start colchicine in the acute phase of gout, within 12 h of the acute-gout attack, while the effect significantly decreased after 36 h [20]. Patients with gout accepting urate-lowering therapy in the acute phase rather than after acute remission might have good prognosis as this approach could shorten the time of serum uric acid reaching the target value and could reduce the risk of chronic kidney injury [19]. Therefore, our findings provided experimental evidence of medication time.

Our study also focused on the unique inflammatory reactions of different synovial cells in gout. This is the first time that the significant proinflammatory role of the sublining FLS in acute gout has been demonstrated. Specifically, sublining FLS were found to be more capable of MSU phagocytosis and caused more severe inflammation than lining FLS. It has been reported that the synovial lining layer controls cellular and molecular transport between the synovial membrane and the joint cavity, maintains joint integrity, and regulates the composition of the synovial fluid [21–24]. In contrast, the sublining layer is a loose connective tissue that contains blood and lymphatic vessels, ensuring efficient transport of nutrients and cells from the systemic circulation to the joints. Previous studies have emphasized the critical role of synovial remodeling in driving arthritis pathology [25,26]. A more proinflammatory function attributed to the sublining FLS was implied [27–30], which aligns with our study. Furthermore, the underlying mechanism of fibroblast-mediated tissue inflammation may be therapeutically targetable. A diphtheria toxin system was used to selectively deplete a pathogenic population (fibroblast activation protein α-expressing fibroblasts), leading to attenuated synovial inflammation and joint damage in experimental arthritis in mice [27]. Therefore, for gout patients with intra-articular MSU crystal deposits, not only should urate-lowering and anti-inflammatory drugs be taken orally, but medication treatment targeting sublining FLS should also be considered.

Looking ahead, there are several areas for future improvements and opportunities. First, a human 3D chip-based chondro-synovial coculture joint model could be used to simulate the pathological process of gout in the whole joint [31]. Furthermore, rather than the in vitro organoid experiments performed in this study, future efforts may be directed toward the in vivo studies to dynamically display gout attacks. Moreover, an experiment where the drug is actually administered should be conducted in vivo to further confirm the drug administration time window. Finally, our work did not comprise MSU deposition data related to other organoids such as aorta/arteries and/or renal organoids. This could represent a direction for future multisite exploration of gout attack.

In summary, our study was the first to simulate the dynamic crystal deposition processes and observe the spatiotemporal interaction between MSU crystals and human synovial organoids. We discovered that the 12-h mark may be the critical window for MSU deposition in the lining layer of synovium. More importantly, we identified a proinflammatory role of sublining FLS in gout, indicating a need for targeted treatment in the future. Our research offers potential guidance for the timing of medication administration, spatiotemporal indications for medication, and targeted therapies.

## Materials and Methods

### Experimental setup and data acquisition

SRS experiments were performed on our home-built system. A commercial femtosecond optical parametric oscillator (Insight DS+, Newport Inc.) generated the source pump (tunable at 680 to 1,300 nm, ~150 fs) and stokes beams (fixed at 1,040 nm, ~200 fs). After passing 2 SF57 glass rods, the 2 femtosecond laser beams were chirped to picoseconds (~3.8 ps for the pump pulse and ~1.8 ps for the stokes). This process enabled us to achieve hyperspectral SRS capability with matched group velocity dispersion. We acquired spectral information by scanning the time delay between the pump and stokes pulses using a delay line [32,33]. The pump and stokes beams were then combined and introduced into a laser-scanning microscope (FV1200, Olympus). The beams were focused by an objective lens (UPLSAPO 25XWMP2, numerical aperture [NA] = 1.0, Olympus) and collected by a high-NA condenser lens (oil immersion, NA = 1.4, Nikon). The SRS process occurred when the pump and stokes beams interacted with the sample simultaneously at the matched Raman frequency, providing highly specific chemical contrast. The stimulated Raman loss (SRL) signal was collected by a photodiode after passing through a band-pass filter (Chroma, ET890/220M). The stokes beam was modulated by an electro-optical modulator (Thorlabs, EO-AM-R-20-C2) at approximately 20 MHz, and the SRL signal was demodulated by a lock-in amplifier (Zurich Instruments, HF2LI). To achieve 3D imaging capability, the objective lens was moved vertically by a piezo actuator. This setup enabled a typical FoV of about 597 × 597 × 900 μm^3^, with the vertical spacing of images set to 3 μm. In this study, we used 2 pump-stokes wavelength combinations for organoid imaging: 802 + 1,040 nm for lipid/protein imaging to depict cells and 977 + 1,040 nm for imaging MSU crystals.

### Isolation and culture of FLS

The isolation and culturing of human FLS were performed as described previously [3]. Synovial tissues were obtained from patients (*n* = 10) with cruciate ligament and meniscus injuries and without hyperuricemia, approved by the Ethics Committee of Huashan Hospital, Shanghai, China (Approval Number KY2023-807) (Table S1). FLS suspensions were prepared by pulverizing synovial tissue, followed by digestion with 2 mg/ml collagenase type I (Sigma-Aldrich) in Dulbecco’s modified Eagle’s medium (DMEM) (Gibco) at 37 °C for 1 to 2 h. The resulting cell suspensions were passed through a 100-μm cell strainer (Gibco) and cultured in tissue culture flasks (Gibco) supplemented with 10% fetal bovine serum (Gibco) and 1% each of penicillin and streptomycin antibiotics.

### Organoid cultivation

We constructed 3D synovial organs (*n* = 30) following a previously described method [21]. FLS were suspended in a Matrigel matrix (BD Biosciences) at a density of 1 × 10^6^ cells/ml. Droplets of the FLS suspension were plated in wells coated with poly-2-hydroxyethylmethaacrylate (Sigma-Aldrich) at 1 ml/well. The Matrigel matrix was allowed to gel for 30 min at 37 °C. Subsequently, the FLS were cultured in DMEM/F12 (Gibco) supplemented with 10% fetal bovine serum and 1% penicillin–streptomycin at 5% CO_2_ and 37 °C for 3 weeks (Fig. S5).

### MSU preparation

MSU crystals were prepared using uric acid (Sigma-Aldrich) according to a previously described protocol [34,35]. In brief, 1 g of uric acid was dissolved in 200 ml of distilled water, followed by the addition of 450 mg of NaOH and heating to 100 °C. The pH of the solution was then adjusted to approximately 7.2 with HCl. The MSU crystals obtained were sterilized by autoclaving for subsequent cell experiments and then dried in an oven. All reagents were prepared under pyrogen-free conditions. These MSU crystals were used to prepare a solution at a concentration of 100 μg/ml with DMEM culture solution. The MSU crystal solutions were added to synovial organoids at various time points (0, 6, 12, 24, and 48 h). Before undergoing SRS microscopy, the organoids were gently washed to remove any extraneous material and leave only the MSU crystals within the organoids.

### Image data processing

We utilized the open-source software ImageJ [33] to delineate the lining and sublining areas within the synovium organoid. Initially, the background of each image for the 3 chemicals was removed by subtracting the corresponding off-resonant images. Subsequently, masks representing organoid tissue, cells, the ECM, the lining, and sublining regions were identified. Organoid areas were manually selected, while cell areas were distinguished by the background and the signal in the lipid channel using the “Find Edges” function. The ECM areas were obtained by subtracting the cell areas from the organoid areas. Lining and sublining areas were distinguished based on cell position and density, with the outer denser layer recognized as the lining. These extracted areas were converted into binary masks for further processing.

Additionally, the 3D image stack of the MSU within the organoid (applying the organoid mask to the MSU channel) was converted to a side view using the “Reslice” function. After applying a “Threshold” to select the MSU crystals, indices indicating MSU deposition depth were derived using the “Analyze Particles” function. The deposition depth value is a median deposition depth over all visible MSU crystals in the images. Furthermore, the length, aspect ratio, area, and intensity of MSU crystals within the organoid, cell, ECM, lining, and sublining on corresponding image stacks could also be derived. The MSU intensity thresholds were set uniformly for the 5 regions, and intensity density was defined to indicate MSU crystals in units of volumes. For normalization, all values except length and aspect ratio of MSU crystals were subtracted by the values of 0 h.

$$\begin{array}{c} \text{Deposition intensity density of}\;\text{MSU}\;\text{crystals}\\ =\frac{\sum \text{MSU}\;\text{intensity}}{\text{areas of each region}\;*\;\text{vertical spacing}} \end{array}$$

$$\begin{array}{c} \text{Phagocytosis intensity density of}\;\text{MSU}\;\text{crystals}\\ =\frac{\sum \text{intracellular}\;\text{MSU}\;\text{intensity}}{\text{areas of cells}\;*\;\text{vertical spacing}} \end{array}$$

We then analyzed the deformation of synovial cells, obtaining their roundness and area using the “Analyze Particles” function. Subsequently, 3D reconstructions were conducted using ImageJ and MATLAB. Statistical analyses were performed on the quantitative indices obtained from image stacks using GraphPad Prism 8.4.0 (GraphPad Software). *t* Tests were employed to assess whether statistically significant differences existed between groups (*P < 0.05, **P < 0.01, and ***P < 0.001).

### Histology and IHC

All synovial organoids underwent fixation with 4% paraformaldehyde in phosphate-buffered saline, followed by embedding in paraffin. Subsequently, the organoids were sectioned and stained with HE and reticulin stains using the Gomori silver impregnation technique as previously described to determine morphology [21]. Additionally, organoid sections were deparaffinized, rehydrated, and antigen-unmasked in citrate buffer. Subsequent steps included incubation with 3% hydrogen peroxide and permeabilization with 0.1% Triton X-100 in phosphate-buffered saline. Sections were then treated with an antibody specific for lubricin, followed by incubation with a secondary antibody. Light microscopic images were captured using a Zeiss Axio observer microscope, and image processing was performed using Adobe Photoshop software.

### Immunofluorescence assay

Synovial organoids were fixed in 4% paraformaldehyde in phosphate-buffered saline. Sections were deparaffinized in xylene, hydrated with decreasing concentrations of ethanol, permeabilized with 0.3% Triton X-100 for 5 min, and blocked with bovine serum albumin for 1 h at room temperature. Primary antibodies against IL-1β and TNF-α were then applied overnight at 4 °C. Confocal laser fluorescence microscopy (Olympus) was used to image the sections. Finally, fluorescence intensity was quantified by observers blinded to sample groups using ImageJ.

## Ethical Approval

All patients critically read and signed the informed consent form (KY2023-807), which was approved by the Ethics Committee of Huashan Hospital. The research followed the guidelines of the 1975 Declaration of Helsinki.

## Data Availability

All data is available in the main text or the supplementary materials.
