# Supplementary material for: Spatiotemporal Observation of Monosodium Urate Crystals Deposition in Synovial Organoids Using Label-Free Stimulated Raman Scattering
Source: Research (Wash D C). 2024 May 27;7:0373. doi: 10.34133/research.0373 (PMC11128648; doi:10.34133/research.0373)
Supplement: Supplementary 1 — Figs. S1 to S5 Table S1 [file research.0373.f1.docx]

**Supplemental Information**

**Figure S1. Spatial resolution.** (A) Line profile and the corresponding 1st derivation. See from the fitting parameter, a spatial resolution of 578 nm was achieved. (B) SRS image of CH stretching bond of dodecane, the experimental condition was the same as organoid and MSU. Yellow line indicates the position to take the line profile. FOV: 211.97*211.97 μm2.


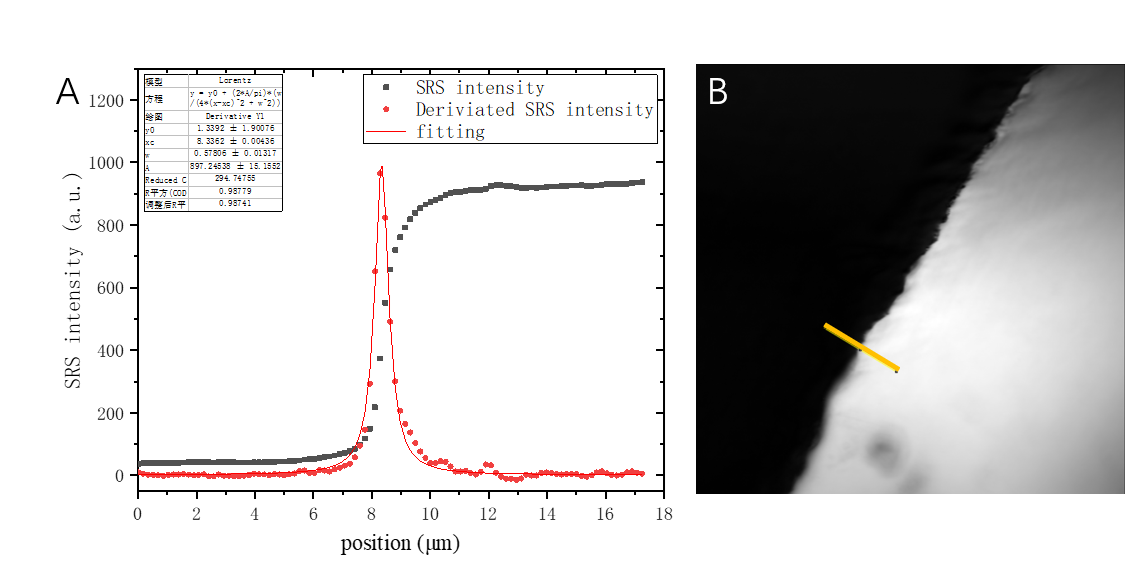


**Figure S2. Spontaneous Raman and stimulated Raman spectra.** (A) Spontaneous Raman and (B) Stimulated Raman spectra the longitudinal transverse optical (LTO) phonon mode of diamond.


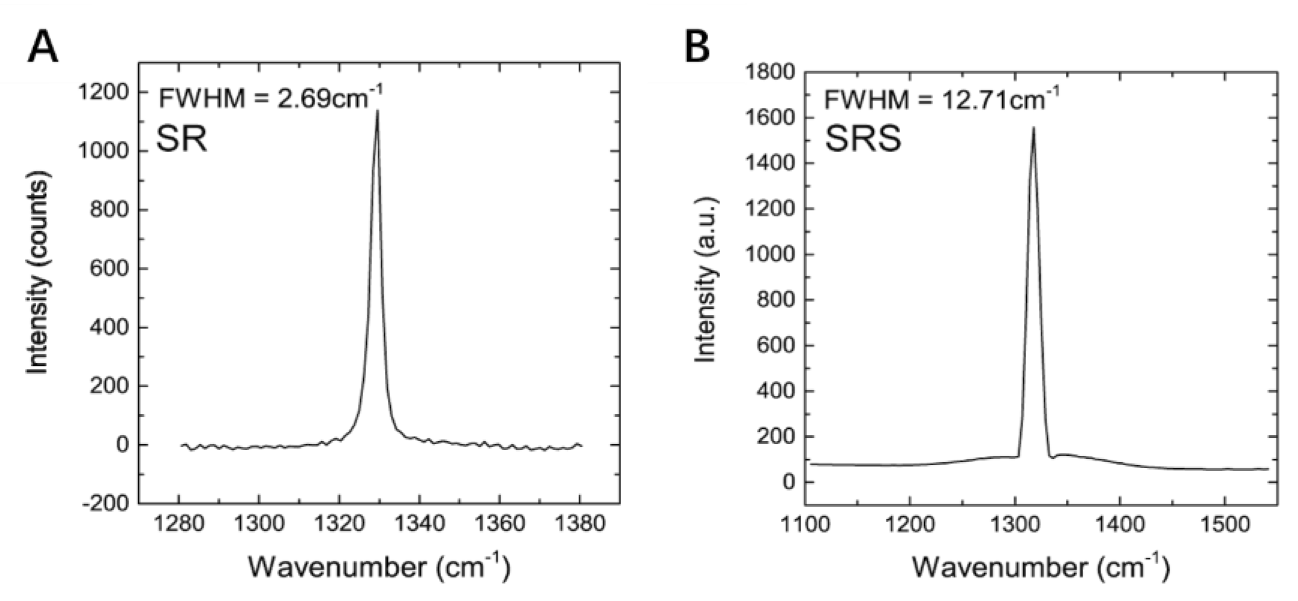


**Figure S3. Demonstration of the spectral bandwidth.** Because of the limited bandwidth of our laser pulses, the spectral focusing mode only cover ~200 cm-1 with fixed beam wavelengths. To cover the broad spectral range of OH stretching from about 3000 to 3500 cm-1, spectral stitching was applied with multiple pump center wavelengths. To made a complete and correct spectra recovery, we chose the pump wavelength not corresponding to the OH vibration modes, but with a approximately equal wave number intervals. 768, 775, 778, 784, and 788 wavelengths corresponded to the central wave numbers of 3405, 3288, 3238, 3140, and 3075 cm-1, respectively.





**Figure S4. 3D time series SRS images of synovial organoids stimulated by MSU crystals.** Representative stereographic side views and top views of human normal synovial organoids after adding MSU crystals for 0, 6, 12, 24 and 48 hours. MSU crystals (yellow, 630 cm-1), lipid (green, 2930 cm-1) and protein (blue, 2930 cm-1).


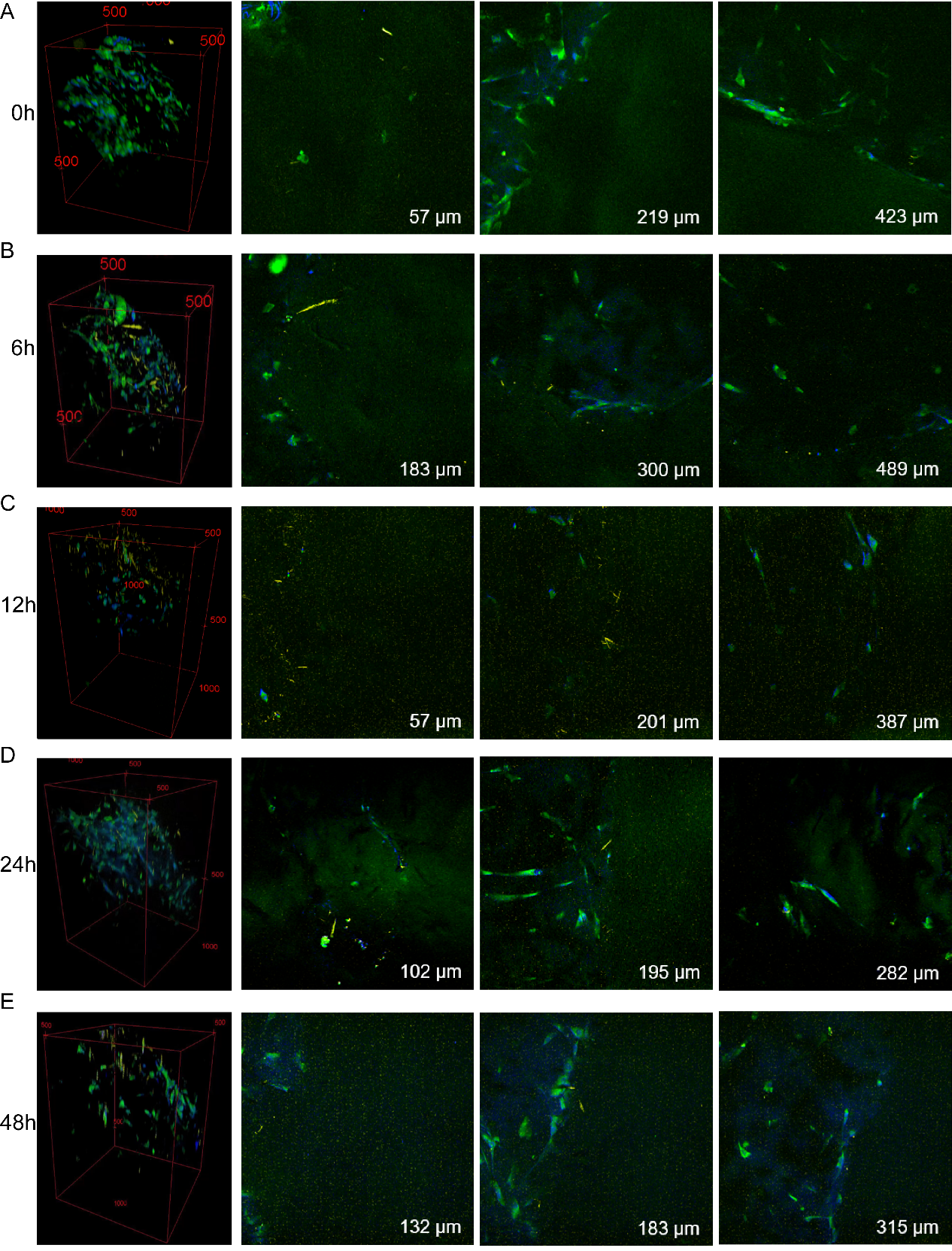


**Figure S5. Schematic of synovial organoid culture and engineering.** Briefly, human synovial tissues from patients with cruciate ligament injuries and meniscus injury were obtained. FLS suspensions were prepared by pulverization of human synovial tissue, followed by digestion with 2mg/ml collagenase type Iin Dulbecco’s modified Eagle’s mediumat 37°C for 1-2 hours. The cell suspensions were then passed through a 100-μm cell strainer and cultured in tissue culture flasks, and then FLS were resuspended in Matrigel Matrix. Droplets of FLS suspension was plated in 1 ml/well coated with Poly-2-hydroxyethylmethaacrylate. The Matrigel Matrix was allowed to gel for 30 min at 37°C. Subsequently, FLS were maintained in culture in DMEM/F12 supplemented with 10% FBS and 1% penicillin-streptomycin at 5% CO2, 37°C for 3 weeks.


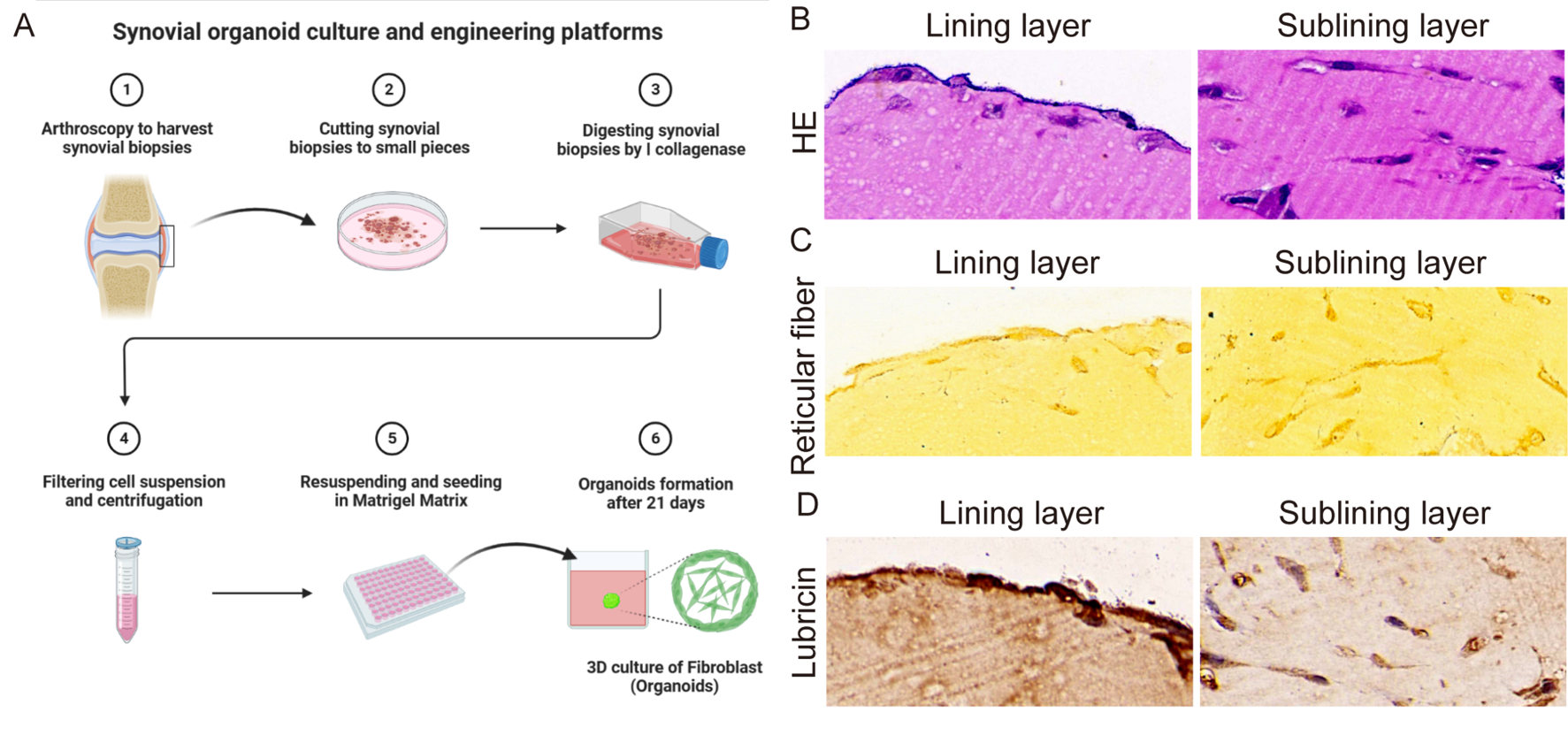


| Year (y) | Gender | Diagnosis | Uric acid concentration (mmol/L) |
| --- | --- | --- | --- |
| 23 | female | Rupture of the anterior cruciate ligament of the right knee | 0.253 |
| 20 | male | Rupture of the anterior cruciate ligament of the right knee | 0.308 |
| 25 | female | Meniscus injury of the right knee | 0.217 |
| 29 | female | Rupture of the anterior cruciate ligament of the right knee | 0.331 |
| 31 | male | Meniscus injury of the right knee | 0.281 |
| 27 | male | Meniscus injury of the right knee | 0.329 |
| 26 | female | Rupture of the anterior cruciate ligament of the right knee | 0.297 |
| 28 | male | Rupture of the anterior cruciate ligament of the right knee | 0.378 |
| 22 | female | Meniscus injury of the right knee | 0.352 |
| 27 | male | Rupture of the anterior cruciate ligament of the right knee | 0.263 |

**Table 1 Basic characteristics of surgical patients**
